# Exploring Ectoine Production From Methanol, Formate, and Electrochemically Produced Formate by *Methyloligella halotolerans*

**DOI:** 10.1002/elsc.70063

**Published:** 2026-01-09

**Authors:** Aykut Kas, Paniz Izadi, Claudius Lenz, Thore Rohwerder, Jens Olaf Krömer, Falk Harnisch

**Affiliations:** ^1^ Department of Microbial Biotechnology Helmholtz‐Centre For Environmental Research—UFZ Leipzig Germany

**Keywords:** C_1_‐substrates, ectoine, electrochemical CO_2_ reduction, formate utilization, *Methyloligella halotolerans*

## Abstract

Microbial synthesis using renewable C_1_‐carbon sources like electrochemically produced formate (e‐formate) represents a promising approach for climate‐neutral chemical production. This study investigates formate utilization for ectoine biosynthesis by the halophilic methylotroph *Methyloligella halotolerans*. Preliminary growth assays confirmed formate utilization using 15–20 mM formate as the sole energy source substrate, when supplemented with yeast extract or vitamin solutions in a mineral salt medium. In a systematic study for ectoine production, formate utilization reached 0.305 ± 0.020 mmol d^−1^ at 20 mM. With different C_1_‐substrates at 20 mM (3 mmol), ectoine production reached 10.3 ± 3.2 µmol (from methanol), 6.5 ± 0.8 µmol (from equimolar methanol/formate mix), 4.4 ± 0.1 µmol (from formate), and 1.2 ± 0.1 µmol (from e‐formate). Medium buffering, pH stability and toxicity limited performance when formate and e‐formate were supplied. Although ectoine yields were suboptimal, the feasibility of e‐formate‐based ectoine biosynthesis under high‐salinity conditions with 9% NaCl, as shown in this study, discloses the great potential for integrating highly efficient electrochemical CO_2_ reduction in saline media with microbial synthesis of organic chemicals.

**Abbreviations**
CDW: cell dry weight
CE: Coulombic efficiency
eCO_2_RR: electrochemical CO_2_ reduction reaction
e‐formate: electrochemically produced formate
GDE: gas diffusion electrode
HPLC: high performance liquid chromatography
ISR: inoculum‐to‐substrate ratio
OD_600_
: optical density at 600 nm

## 1 Introduction

Climate‐neutral production of chemicals in a circular economy requires shifting away from fossil carbon sources toward renewable alternatives. Among the emerging strategies, the use of CO_2_ as a carbon feedstock has gained significant attention [1]. However, CO_2_ is thermodynamically stable and requires energy input for its activation and conversion into more reduced, biologically accessible carbon compounds [2]. Electrochemical CO_2_ reduction (eCO_2_RR) provides a sustainable approach to convert CO_2_ using (renewable) electric energy [3]. Among the possible products, formate is suggested as a promising intermediate for integrating with microbial synthesis by serving as C_1_‐substrate. This is due to the high solubility of formate in water, and high selectivity and low overpotential of eCO_2_RR to formate [4]. Microbial synthesis of value‐added products from formate has gained increasing interest, for instance, through metabolic engineering of *Escherichia coli* [5, 6]. For combining eCO_2_RR with microbial synthesis using *Cupriavidus necator* [7, 8], *Methylobacterium extorquens* AM1 [9, 10] and acetogens [11, 12] have been explored for the production of bioplastics, longer chain carboxylic acids, amino acids, and alcohols. However, these efforts have largely focused on bulk or intermediate‐value compounds (e.g., acetate, ethanol, butanol, polyhydroxyalkanoates, etc.), and the potential for the synthesis of high‐value products remains underexplored.

Hypersaline environments present substantial challenges to conventional microbial production systems due to osmotic stress, reduced water activity, and ionic toxicity. However, halophilic microorganisms are well adapted to thrive under such conditions, with reported salt tolerances reaching up to 35% NaCl [13]. Halophiles possess unique metabolic features, including compatible solute accumulation, salt‐adapted enzymes, specialized membrane structures, and efficient ion transport systems [14]. As high‐salt environments can be beneficial for eCO_2_RR under biocompatible conditions, it is setting the stage for coupling with halophilic microbial synthesis. For instance, eCO_2_RR in saline media with salt concentrations up to 17% NaCl achieved energy efficiency of 28.2%, representing an almost twofold improvement over non‐saline buffer‐only conditions, benefiting from increased ionic conductivity and reduced ohmic losses [15]. Among the halophiles, *Methyloligella halotolerans* is a moderate halophile recently described to grow in high‐salt media up to 8.8% NaCl and synthesize ectoine from the C_1_‐substrate methanol [16]. Ectoine is a compatible solute produced to counterbalance osmotic stress that has become a target compound for biotechnological production [17]. Ectoine holds exceptional market value, around 1000 USD kg^−1^ [18], due to its protective roles in cosmetics, medicine, and biotechnology, while formate itself has a value of around ∼1 USD kg^−1^ [19]. These factors make ectoine an attractive model compound for combining eCO_2_RR with microbial synthesis at saline conditions. While *M. halotolerans* is known to metabolize methanol via the serine cycle [16, 17, 18, 19, 20], its ability to utilize formate as a sole energy source for growth or synthesis of ectoine has not yet been described.

Here, we present a proof‐of‐concept for formate‐based growth and ectoine production by *M. halotolerans*. Therefore, we investigated the ability of *M. halotolerans* to grow on formate as a sole energy source. Subsequently, we compared ectoine production by *M. halotolerans* from four different C_1_‐substrates: methanol, formate, e‐formate (i.e., formate generated via eCO_2_RR at Sn‐based gas diffusion electrodes [15]), and a methanol/formate mix. Thereby, we show that saline microbial media can be used as an electrolyte solution for combined electrochemical‐microbial synthesis of high‐value compounds, providing a proof‐of‐concept contribution to the ongoing development of electrobiorefineries [21].

## 2 Materials and Methods

### 2.1 Preliminary Growth on Formate With *M. halotolerans*

Cultivation of *M. halotolerans* (DSM 25045) was carried out in modified Choi [22] mineral salt medium adapted for high‐salinity conditions. The medium consisted of standard Choi components supplemented with 90 g/L NaCl, a defined trace element solution, and either 0.2 g/L yeast extract or a modified DSMZ 141 vitamin solution (100‐fold stock) containing an additional 5 mg/L cyanocobalamin (vitamin B_12_).

Cultures were initially grown in 100‐mL baffled Erlenmeyer flasks containing 50 mL of medium. Incubation was performed at 30°C in an orbital shaker (INFORS HT Multitron AJ125, Infors AG, Switzerland) set to 150 rpm. Cryostocks of *M. halotolerans* were initially used for inoculation to assess growth and viability under conditions similar to those described previously for growth and isolation of the strain with 0.5% (v/v) methanol (≈125 mM) [16]. For initial cultivation from cryostocks, 100 mM methanol was used as the primary carbon source, supplemented with 15 mM formate to promote adaptation to formate utilization in later stages. Subsequent experiments evaluated growth with formate as a sole carbon source (15–20 mM), supplemented either with yeast extract or DSMZ 141 vitamin solution. All cultivation steps, including inoculation and sampling, were carried out under sterile conditions in a Class II biological safety cabinet (Herasafe KS 15, Thermo Scientific Heraeus, Germany). Different inoculum‐to‐substrate ratios (ISR) were tested during preliminary growth experiments to assess the potential for sustaining subculturing and growth. A detailed description of the growth media and ISR conditions is provided in Supporting Information Section S1.1 and S1.2.

### 2.2 Ectoine Production From C_1_‐substrates

For the systematic study on ectoine production, *M. halotolerans* was cultivated in modified Choi medium supplemented with the modified DSMZ 141 vitamin solution containing 50 mg/L cyanocobalamin (vitamin B_12_). Four C_1_‐substrates were tested at a total concentration of 20 mM: methanol, formate, e‐formate, and a methanol/formate co‐substrate mix (10 mM each). A concentration of 20 mM was selected, as initial tests showed this concentration led to the best microbial growth (Supporting Information Section S2.1). All experiments were conducted in 300‐mL Erlenmeyer flasks with 150 mL volume of liquid medium, incubated at 30°C with a shaking incubator (INFORS HT Multitron AJ125, Infors AG, Switzerland) set at 150 rpm. To initiate the experiment with a high biomass concentration, precultures were grown in 100 mM methanol and 20 mM formate. Prior to inoculation of the experimental setups and the second feeding of substrates on Day 15 of cultivation (for methanol, formate and co‐substrate conditions), complete consumption of both substrates was confirmed by high performance liquid chromatography (HPLC). Biological triplicates were performed for each condition.

### 2.3 Electrochemical Formate (e‐formate) Production

The eCO_2_RR to formate was carried out in a custom‐built flow‐through reactor [7] as described before [15], equipped with a Sn‐based gas diffusion electrode (GDE) (Gaskatel GmbH, Germany) as the cathode and a platinum foil as the anode. A proton exchange membrane (Nafion 117, DuPont, USA) was used to separate the cathode and anode compartments. Both chambers were filled with modified Choi medium to minimize cross‐over of ions, which is a change in media composition for subsequent microbial cultivation. The resulting electrolyzed medium containing formate from the cathode was collected and directly used as e‐formate for ectoine production. A detailed description of the reactor setup and operation is provided in Supporting Information Section S1.3.

### 2.4 Sampling and Analytical Methods

In preliminary tests, 2 mL samples were collected to monitor OD_600_, pH, and substrate concentrations. In ectoine production experiments, 8 mL samples were taken every 5 days for the aforementioned measurements, with 2 mL additionally used for ectoine extraction. High‐frequent sampling was avoided to minimize disturbance due to slow growth. For electrolysis experiments with e‐formate, 4 mL catholyte samples were withdrawn at 30‐min intervals. Prior to sampling, the lines were rinsed with 2 mL of catholyte.

All C_1_‐substrates (formate, e‐formate, and methanol) were quantified using HPLC (Prominence HPLC, Shimadzu Scientific Instruments, Japan), while ectoine was analyzed by ultra‐high performance liquid chromatography coupled to mass spectrometry (UHPLC‐MS) (Vanquish UHPLC with Exploris 240 MS, Thermo Fisher Scientific, Germany).

Ectoine extraction involved methanol‐based cell lysis, followed by evaporation and reconstitution in water, adapted from the described methods of ectoine and metabolite extractions [23, 24], followed by centrifugation and dilution with acetonitrile prior to analysis. Ion suppression was observed with UHPLC‐MS measurements and evaluated by spiking ectoine standards into pure solvent and culture extracts (t0 and t5), allowing calculation of a correction factor based on relative signal increases. Hydroxyectoine, a derivative of ectoine and an interchangeable compatible solute commonly produced under salt stress, was also considered as a possible product [25]; however, it was not detected in any of the samples. A detailed description of substrate and ectoine quantification methods, and gas analysis procedures is provided in Supporting Information Section S1.4.

### 2.5 Data Processing and Calculations

Formate and methanol are reported in absolute amounts (i.e., moles) rather than titer to account for evaporation‐induced volume and concentration changes. Both water evaporation and substrate volatilization were monitored throughout the measurement with abiotic controls, as they significantly influenced substrate concentration and apparent consumption under aerobic shake flask conditions using cellulose caps (i.e., abiotic controls). Evaporation was estimated from abiotic controls during preculture experiments, revealing an average loss of approximately 1 mL per day for an initial volume of 150 mL. To correct for this, substrate‐specific evaporation rates were derived from abiotic controls using linear regression: formate showed a daily loss of 1.18% (that was also accounted for e‐formate), while methanol showed a 3.18% per day from their initial molar amounts.

Substrate specific ectoine yields ($\eta_{ectoine/formate}$, $\;\eta_{ectoine/methanol}$, mmol mol^−1^) were then determined by evaluating the ectoine content increase over the 5‐day period preceding the observed maximum. For each replicate, the amount of substrate consumed during this interval was calculated individually, incorporating the corrected evaporation and volatilization losses. A detailed description of yield calculations with substrate‐specific abiotic losses, as well as Coulombic efficiency (CE) calculations and electrochemical product quantification, is provided in Supporting Information Section S1.5.

## 3 Results and Discussion

### 3.1 Preliminary Growth on Formate With *M. halotolerans*

*M. halotolerans* utilizes the serine cycle for assimilation of C_1_‐substrates, and its ability to grow on methanol has been previously described [15, 16, 17, 18, 19, 20, 21, 22, 23, 24, 25, 26]. Key enzymes of the serine pathway have been identified in its annotated genome sequence [20], supporting the metabolic potential for methanol‐ and formate‐based growth. Since both methanol and formate can be assimilated by the serine cycle, it was expected to serve as a utilizable carbon and energy source. However, the extent to which formate can be used for growth under saline conditions remained unclear and was therefore assessed in a series of shake flask experiments. To evaluate growth performance and avoid diluting the inoculum in conditions of limited growth, a range of inoculum‐to‐substrate ratios (ISR) was initially tested using 15 to 20 mM formate.

Both yeast extract and vitamin‐supplemented cultures showed comparable trends (Figure S1) in formate consumption and growth, suggesting that chemically defined media are sufficient for cultivation under the tested conditions. This offers advantages for process integration with electrochemical synthesis, as yeast extract was found to interfere with eCO_2_RR, likely due to redox‐active components or electrode fouling reported in previous studies [27]. Notably, formate metabolism led to substantial pH shifts from 6.5 up to 8.3 (Figure S2), emphasizing the need for buffering strategies or controlled pH management in future process designs. Formate utilization rates were quantified for each ISR and supplementation condition to assess the effect of inoculum size and medium composition. The highest formate uptake rates during the initial 5 days were observed for ISR = 0.16 in both conditions, with yeast extract supplemented cultures reaching 0.140 ± 0.002 mmol d^−1^ and vitamin supplemented cultures 0.142 ± 0.010 mmol d^−1^ (Table S1). Maximum OD_600_ reached (Figure S1) were 0.278 ± 0.130 and 0.202 ± 0.094 for yeast extract and DSMZ 141 vitamin solution supplemented media, respectively, while for reference, methanol at 20 mM led to OD_600_ values ranging from 0.744 to 1.058. This aligns with previous reports describing the inherently lower growth kinetics of methylotrophs utilizing the serine cycle compared to those operating via the Ribulose Monophosphate (RuMP) cycle when utilizing methanol [28]. Notably, organisms using RuMP cycle cannot assimilate formate, as they lack the capacity to reduce it to formaldehyde. However, when methylotrophs use the serine cycle, formate reduction to formaldehyde imposes an additional energetic burden and potential toxicity, leading to greater physiological stress than methanol‐based growth [29]. A detailed description of initial subculturing, growth inhibition thresholds, and semi‐continuous feeding strategies is provided in Supporting Information Section S2.1. The reduced growth efficiency on formate is attributable to its lower degree of reduction (2 e^−^ per C) compared to biomass (4.2 e^−^ per C, [30]), whereas methanol is more reduced (6 e^−^ per C). As a result, formate provides fewer reducing equivalents and additionally requires ATP‐dependent reduction to carrier‐bound formaldehyde in the serine cycle. In contrast, methanol delivers surplus reducing equivalents that offset this activation cost and thereby supports higher growth yields. This aligns with observed molar biomass yields, which were two‐ to three‐fold lower on formate (3.3–6.9 gmol^−1^) than on methanol (9.8–13.1 gmol^−1^) for serine pathway‐utilizing bacteria grown in carbon‐limited continuous cultures [31].

### 3.2 Ectoine Production With C_1_‐Substrates

To evaluate ectoine biosynthesis under halophilic conditions, *M. halotolerans* was cultivated in modified Choi medium supplemented separately with formate, methanol, e‐formate (discussed in detail in Section 3.3), and equimolar methanol/formate mix as the sole energy source. Ectoine formation from different C_1_‐substrates was performed in order to demonstrate that e‐formate can be functionally connected to fine‐chemical production under high‐salinity conditions and thus provide a proof‐of‐concept for this specific kind of an electrobiosynthesis. Ectoine production was observed for the use of all substrates, while the inoculum control without a carbon substrate showed a consistent decline in ectoine content across all time points (Table S2). This decline aligns with observations that microorganisms also catabolize osmoprotectant compounds like ectoine [32], supporting the notion that ectoine is metabolized under nutrient‐limited or reduced salt stress conditions.

**FIGURE 1:**
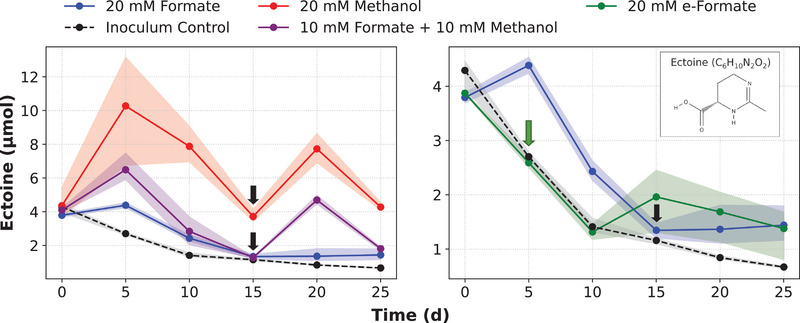
Time course of ectoine production by *M. halotolerans* using different substrates. (A) Comparison of 20 mM formate, 20 mM methanol, 10 mM formate + 10 mM methanol and inoculum control, (B) expanded scale for formate, e‐formate (20 mM) and inoculum control (*n* = 3). Black arrows show the second feed for formate, methanol and co‐substrate conditions, green arrow shows the time point when buffer addition and pH adjustment were done for e‐formate set (Section 3.3 for details). Corresponding C_1_‐substrate concentration profiles are shown in Figure S3, and net ectoine changes over 5‐day intervals are summarized in Table S2. The inset shows the molecular structure of ectoine.

The highest ectoine amount of 10.3 ± 3.2 µmol was achieved (Figure 1A) after 5 days of first feeding with 20 mM (i.e., 3 mol) methanol, which is in agreement with the methanol‐based synthesis capacities of this strain reported previously [16]. Co‐substrate feeding (10 mM formate + 10 mM methanol) resulted in ectoine content of 6.5 ± 0.8 µmol. Feed with only 20 mM formate led to lower but still net positive ectoine production of 4.4 ± 0.1 µmol when compared to the inoculum control at the same time point, showing 2.7 ± 0.1 µmol. This confirms a limited but functional capacity of *M. halotolerans* to synthesize ectoine from solely formate. A second substrate feeding on day 15 of the first inoculation led to a statistically significant increase in ectoine content, in both, the methanol (Δ = 4.01 ±  0.50 µmol, *p* = 0.0052) and co‐substrate (Δ = 3.41 ± 0.28 µmol, *p* = 0.0022) fed conditions, based on the ectoine content at the time of the second feed. In contrast, the formate‐fed culture showed no significant change (Δ = 0.02 ± 0.41 µmol, *p* = 0.944). Meanwhile, the inoculum control (i.e., without addition of substrate) exhibited a statistically significant decrease for the same time period (Δ = –0.32 ± 0.04 µmol, *p* = 0.0063).

C_1_‐substrate utilization was monitored (Figure S3), and utilization rates, even when corrected for evaporation and volatilization, were higher than in preliminary tests. In the preliminary test, low‐OD_600_ precultures maintained solely on formate were used to exclude external influences and confirm the feasibility of formate utilization. For ectoine production, a denser preculture (OD_600_ ≈ 1.3) was applied, which supported faster uptake and allowed comparison of C_1_‐substrate utilization rates. Notably, the co‐substrate condition showed the maximum uptake rate of 0.510 ± 0.008 mmol d^−1^ compared to 0.406 ± 0.003 mmol d^−1^ for methanol, 0.305 ± 0.020 mmol d^−1^ for formate and 0.124 ± 0.057 mmol d^−1^ for e‐formate.

Substrate‐specific ectoine yields ($\eta_{ectoine/substrate}$, mmol mol^−1^) revealed that only a minor fraction of the available C_1_‐substrate was utilized toward ectoine biosynthesis, with the majority likely used for cell growth and maintenance. The highest yield for ectoine production was observed with 20 mM methanol, reaching 2.91 ± 1.24 mmol mol^−1^ in the first feed and 3.02 ± 0.33 mmol mol^−1^ in the second feed. Co‐substrate feeding (10 mM formate + 10 mM methanol) resulted in 0.94 ± 0.32 mmol mol^−1^ and 1.95 ± 0.21 mmol mol^−1^, respectively, while 20 mM formate alone showed the lowest yields with 0.39 ± 0.07 mmol mol^−1^ and 0.33 ± 0.21 mmol mol^−1^ over the two feeding cycles (Figure S4). Even when the preculture was grown on a concentration of C_1_‐substrates being six times higher, comprising 100 mM methanol and 20 mM formate, ectoine yield remained low at 25.7 ± 5.14 mmol mol^−1^. Ectoine production is strongly influenced by salinity, temperature, and cultivation strategy, with reported yields in other pure culture halophilic strains (Table 1) higher than those obtained here. With these systems, substrate levels exceed the amounts required for basic growth and maintenance, allowing additional carbon and energy to be directed toward ectoine biosynthesis. In contrast, for instance, 20 mM formate only provides limited surplus, and further increases in formate supply are constrained by toxicity and pH effects, underscoring the need to mitigate formate‐related stress factors to achieve higher ectoine contents. Nevertheless, ectoine yields normalized to carbon input (mc‐mol c‐mol^−1^) are comparable to high‐performing strains, especially under preculture conditions with adequate C_1_‐substrate (120 mM in total), indicating efficient carbon channeling despite lower volumetric yields. The lower energetic yield of formate can additionally limit ectoine production, which is known to be a highly energy‐intensive process, for example, 1 mol ectoine is equivalent to about 40 mol ATP in a glucose‐based aerobic metabolism [33].

**TABLE 1:** Substrate‐specific ectoine yields ($\eta_{ectoine/substrate}$, mmol mol^−1^) with pure culture halophilic strains under different substrate and salinity conditions.

Strain	Substrate^a^	Concentration (mM)	Salinity (% NaCl)	Temp (°C)	Max yield (mmol mol^−1^)	Max yield normalized to C (mc‐mol c‐mol^−1^)	Reference
Halomonas elongata DSM142	Glc	111	15.0	25	139.4	139.4	[24]
Brevibacterium epidermis DSM20659	Glu	296	5.9	30	59.5	71.4	[34]
Halomonas salina DSM5928T	Glu	1183	2.9	33	166.5	199.8	[35]
Methyloligella halotolerans DSM25045	MeOH + For	100 + 20*	9.0	30	**25.7** ^b^	**154.2±30.6** ^b^	This study
	MeOH	20	9.0	30	**3.0±0.3** ^c^	**18.0±2.0** ^c^	
	MeOH + For	10 + 10	9.0	30	**2.0±0.2**	**12.0±1.3**	
	For	20	9.0	30	**0.4±0.1** ^c^	**2.4±0.4** ^c^	

^a^ Glc, glucose; Glu, glutamate (as monosodium glutamate, MSG); MeOH, methanol; For, formate (as sodium formate).

^b^ Values reflect preculture conditions that used high MeOH to promote higher initial biomass levels.

^c^ Theoretical yields of MeOH and For are 143 mmol mol^−1^ and and 57 mmol mol^−1^, respectively.

The metabolic stoichiometry analysis (Figure S5, Supporting Information S2.2) demonstrates that the synthesis of ectoine from C_1_‐substrates involves multiple metabolic steps, including initial assimilation to key intermediates acetyl‐CoA and oxaloacetate. For formate‐based metabolism, acetyl‐CoA and oxaloacetate formation demands considerable additional oxidation equivalents, thus consuming extra formate molecules (17.5 formate → ectoine + 11.5 CO_2_). In contrast, methanol metabolism benefits from inherent partial oxidation, generating additional reducing equivalents (PQQ and NADH) exchangeable for ATP via the respiratory chain. This reduces the total substrate demand and significantly lowers CO_2_ emissions (7 methanol → ectoine + 1 CO_2_). These steps collectively underscore methanol feed as metabolically advantageous, requiring fewer oxidation reactions, thereby improving carbon efficiency and also minimizing CO_2_ byproduct formation compared to formate‐based ectoine production. This quantitative difference is directly mirrored in the ectoine content (Figure 1), where formate‐fed cultures show a decline below the initial content at t_15_ before the second feeding. Approximately 2.33‐fold more formate (≈46.6 mM) would be required to match the energetic contribution of 20 mM methanol, a concentration between the tested levels (20 mM and 100 mM) but one that may already impose growth‐limiting effects due to formate‐associated stress.

Previously, calorimetric analysis of *Halomonas elongata* at 9.5% NaCl showed ca. 20% of substrate combustion enthalpy was shown to be allocated to ectoine synthesis, while ca. 50% directed toward biomass, highlighting the substantial energetic cost of ectoine as a stress protectant [36]. This was also evident in our study, two successive 20 mM methanol feeds (40 mM total) supported growth to an OD_600_ of 1.06 ± 0.03 (Figure S6), similar to the preculture on 100 mM methanol + 20 mM formate (1.26 ± 0.03), but with an ectoine yield nearly nine‐fold lower. The comparatively low ectoine accumulation in *M. halotolerans*, compared with previously reported literature values [16, 36, 37, 38, 39, 40], is described in Supporting Information Section S2.2. Microorganisms are also capable of producing other compatible solutes in response to osmotic stress, such as the ectoine precursors glutamate and aspartate [41, 42, 43], and other compatible solutes, including glycine betaine, may already accumulate intracellularly under high salt conditions [44, 45]. However, these were not quantified in this study, as the focus was to assess whether formate alone could support complete flux toward ectoine as a high‐value end product.

### 3.3 Electrochemical Formate (e‐Formate) Production and Subsequent Microbial Utilization for Ectoine Synthesis

To provide formate for microbial cultivation under saline conditions using the modified Choi medium, eCO_2_RR to formate was carried out, resulting in an almost linear increase in formate (i.e., e‐formate) concentration over time (*R* ^2^  =  0.996). At 90 min, the process yielded an average of 9.59 ± 0.99 mmol e‐formate, approaching the 20 mM target concentration in the ca. 500 mL electrolyte (Figure S7) used for subsequent feeding to *M. halotolerans* cultures. The final product distribution for the eCO_2_RR included 3.38 ± 0.53 mmol H_2_ and 0.71 ± 0.06 mmol CO as minor side products. Electron balance calculations showed high selectivity toward e‐formate, with CE values of 69.4 ± 7.6% for e‐formate, 24.1 ± 5.0% for H_2_, and 5.1 ± 0.4% for CO at 90 min (Figure S8). While the electron recovery was nearly complete, selectivity toward e‐formate was slightly lower than typically observed in similar setups with buffer‐based systems [8] likely due to the absence of strong buffering and the complex ionic composition of the saline medium. Using the identical setup with a 200 mM phosphate buffer supplemented with 10% NaCl, previously CE for formate was found as 73.1 ± 1.3% and for H_2_ as 9.5 ± 1.3% [15]. The observed H_2_ production as a side reaction may be attributed to trace metals acting as redox‐active compounds present in the complete halophilic growth medium that could enable higher H_2_ evolution rates [46] rather than eCO_2_RR to formate. This trend aligns with previous results using a model halophilic medium, where high salinity likely caused a selectivity shift from eCO_2_RR to formate towards HER [47]. Although high salinity can reduce CO_2_ solubility in aqueous electrolytes [48], the use of GDEs ensured a continuous CO_2_ supply at the catalyst surface. Nevertheless, ion accumulation and hydration effects under these conditions may have altered local pH and ion dynamics [49, 50]. In addition, cation–intermediate interactions [51] and anion adsorption are known to affect interfacial charge distribution and product selectivity [52].

When feeding the e‐formate produced through eCO_2_RR to the *M. halotolerans* shake flask cultures, ectoine content and formate amount were similar to the inoculum control during the first 5 days, with minimal e‐formate consumption (Figure 1B, Figure S3) and a significant pH increase (6.5 ± 0.1 to 7.9 ± 0.1, Figure S9) within this period. Although the medium was adjusted to near‐neutral pH prior to inoculation, these results suggest that the buffering capacity was compromised during the eCO_2_RR process due to consumption of protons for eCO_2_RR and HER that shifted the H_2_PO_4_^−^/ HPO_4_^−^ equilibrium (pK_a_ ≈ 7.2) and drove the pH outside the effective range of the buffer. After restoring phosphate buffering (24.5 mM) and re‐adjusting the pH, e‐formate utilization and ectoine production were observed after 15 days with 2.0 ± 0.6 µmol (inoculum control was at 1.2 ± 0.1 µmol, Figure 1B), however, with delayed kinetics (approximately lacking 10 days). While ectoine production was ultimately confirmed, e‐formate yielded the lowest ectoine content, likely due to both early metabolic inactivity and medium instability as shown by the reduced maximum ectoine content and limited formate utilization compared to other conditions (see Section 3.2) Likely, the performance can be improved under conditions that prevent medium alterations during electrolysis, for example by supplying vitamins and other salts to the e‐formate solution post eCO_2_RR or by employing a coupled one‐pot configuration with active pH control. Additional strategies such as strain engineering could be adopted to improve C_1_‐substrate utilization [53, 54] and manage formate toxicity [6], together with copper‐based electrodes to co‐produce methanol–formate mixtures [55, 56] for further enhancing ectoine yields under saline conditions.

## 4 Concluding Remarks

This study establishes formate as a viable carbon source for ectoine production in the halophilic methylotroph *M. halotolerans*. By using e‐formate for ectoine synthesis, it showcases how saline electrolytes can be used for combined electrochemical‐microbial synthesis of high‐value compounds in electrobiorefineries. As predicted by the energetic constraints, lower yields compared to methanol were achieved, emphasizing the importance of optimizing formate supply, buffering capacity, and microbial robustness under coupled bioelectrosynthesis. Optimization of electrochemical parameters, including improved buffering, electrolyte formulation, and electrode selectivity under high‐salinity environments, could further enhance e‐formate yields. Further advancements through strain engineering, adaptive laboratory evolution, co‐substrate feeding strategies and harvesting ectoine through non‐destructive methods rather than cell lysis could significantly enhance productivity.

## Conflicts of Interest

The authors declare no conflicts of interest.

## Supporting information




**Supporting File 1:** elsc70063‐sup‐0001‐SuppMat.docx.

## Data Availability

The data that support the findings of this study are available from the corresponding author upon reasonable request.
