# Supplementary material for: Exploring Ectoine Production From Methanol, Formate, and Electrochemically Produced Formate by Methyloligella halotolerans
Source: Eng Life Sci. 2026 Jan 9;26(1):e70063. doi: 10.1002/elsc.70063 (PMC12784286; doi:10.1002/elsc.70063)
Supplement: Supplementary file 1 — Supporting File 1: elsc70063‐sup‐0001‐SuppMat.docx. [file ELSC-26-e70063-s001.docx]

**Supporting Information**

*Short Communication*

**Exploring Ectoine Production from Methanol, Formate, and Electrochemically Produced Formate by Methyloligella halotolerans**

Aykut Kas^1^, Paniz Izadi^1^, Claudius Lenz^1^, Thore Rohwerder^1^, Jens Olaf Krömer^1^, Falk Harnisch^1,*^

^1^ Department of Microbial Biotechnology, Helmholtz-Centre for Environmental Research - UFZ, Leipzig, Germany

**Correspondence:** Prof. Dr. Falk Harnisch, [falk.harnisch@ufz.de](mailto:falk.harnisch@ufz.de), Department of Microbial Biotechnology, Helmholtz-Centre for Environmental Research - UFZ, Leipzig, Permoserstrasse 15, 04318 Leipzig, Germany

**Practical Application**

This study demonstrates the integration of electrochemical CO_2_ reduction reaction (eCO_2_RR) with microbial biosynthesis under high‐salinity conditions, specifically focusing on formate‐based production of ectoine, a high‐value biotechnological compound. Using the halophilic methylotroph *Methyloligella halotolerans*, we show that formate from eCO_2_RR can be used for microbial growth and ectoine production despite challenges related to energetic yields, formate toxicity and pH control. Our findings highlight a viable route towards sustainable, climate‐neutral production systems from CO_2_ to ectoine under the umbrella of power‐to‐chemicals. This approach can directly benefit sectors such as biotechnology, cosmetics, and pharmaceuticals, offering a scalable and sustainable pathway for production of high‐value products from renewable carbon sources.

**1. Materials and Methods**

**1.1 Media Composition**

The composition of the modified Choi medium [22] was as follows: KH_2_PO_4_ 1.31 g/L, Na_2_HPO_4_ 2.13 g/L, NaCl 90.0 g/L, (NH_4_)_2_SO_4_ 1.50 g/L, MgSO_4_·7H_2_O 0.45 g/L. Final concentrations of the trace elements were CaCl_2_·2H_2_O 16.7 mg/L, FeSO_4_·7H_2_O 1.30 mg/L, MnSO_4_ 0.10 mg/L, ZnSO_4_·7H_2_O 0.13 mg/L, CoCl_2_·6H_2_O 0.04 mg/L, Na_2_MoO_4_·2H_2_O 0.04 mg/L, CuSO_4_·5H_2_O 0.04 mg/L, and H_3_BO_3_ 0.03 mg/L.

The vitamin concentration in the medium included the following: biotin (B_7_) 0.02 mg/L, folic acid (B_9_) 0.02 mg/L, pyridoxine HCl (B_6_) 0.10 mg/L, thiamine HCl (B_1_) 0.05 mg/L, riboflavin (B_2_) 0.05 mg/L, nicotinic acid 0.05 mg/L, calcium pantothenate (B_5_) 0.05 mg/L, cyanocobalamin (B_12_) 0.05 mg/L, p-aminobenzoic acid 0.05 mg/L, and lipoic acid 0.05 mg/L. The pH was adjusted to 7.0 prior to sterilization.

**1.2 Preliminary Growth Studies**

To assess the feasibility of formate utilization and establish suitable cultivation parameters for M. halotolerans under saline conditions, preliminary shaking-flask experiments were conducted. These tests formed part of an extended substrate screening campaign alongside other halophilic pure cultures (not reported here). Experiments were carried out in duplicate to initially evaluate feasibility, followed by triplicate experiments once formate consumption was confirmed.

To optimize the effective inoculum-to-substrate ratio (ISR) and avoid dilution effects that could further compromise already limited growth, a series of inoculum conditions were tested by varying the biomass concentration while keeping the inoculation volume constant. Cell pellets equivalent to 2–16% of the culture volume were harvested by centrifugation (10000 rpm, 5 min) at the end of the growth phase on day 10 and resuspended in 1 mL fresh medium, corresponding to ISR values from 0.02 to 0.16. This approach was used to ensure that subcultures maintained sufficient biomass input even under weak growth conditions.

To test the limits of growth on formate and circumvent the inhibitory effects of high initial formate concentrations, a semi-continuous feeding strategy was investigated. In this approach, 20 mM formate was added every 10 days, using medium excluding yeast extract and Vitamin 141 solution was added to compensate for evaporation losses and volatilization of other compounds in the media. The pH was maintained near its natural value by adjustment with 0.2 N HCl or 0.2 N NaOH after each feeding.

**1.3 Electrochemical Formate (e-formate) Production**

For producing e-formate, eCO_2_RR was performed following the detailed methodology described previously [15]; briefly, using a Sn-based GDE (Gaskatel GmbH, Germany) as the cathode, electrolyte was circulated continuously at 60 mL min^−1^, and CO₂ gas (99.5%) was supplied at 45 mL min^−1^ in a gas-tight configuration. Galvanostatic operation was applied at −50 mA cm^−2^ for 90 minutes, yielding catholyte formate concentrations of approximately 20 mM matching the preliminary growth studies. Prior to starting eCO_2_RR, the system was equilibrated for 15 to 30 minutes to eliminate leaks and stabilize flow. All electrochemical experiments were conducted at room temperature (23 ± 1 °C) under a fume hood.

**1.4. Sampling and Analytical Methods**

Formate, e-formate and methanol detection was carried out using a HPLC (Prominence HPLC, Shimadzu Scientific Instruments, Japan) equipped with a refractive index detector (RID-20A, Shimadzu) and a Hi-Plex H column (300 mm × 7.7 mm, 8 µm pore size; Agilent Technologies, Germany), along with a Carbo-H pre-column (4 mm × 3 mm). Isocratic elution was performed with 0.005 M H₂SO₄ as the mobile phase at a flow rate of 0.5 mL min⁻¹, column temperature of 50 °C, and a runtime of 30 minutes. External standard calibration was performed for formate with six concentrations (0.65 to 43.0 mM, R² = 0.99) and for methanol with five concentrations (3.3 to 157.7 mM, R² = 0.99). Before HPLC analysis, samples were acidified and diluted with 0.01 M H_2_SO_4_ 1:8 (v/v) for formate and 1:2 (v/v) for methanol and filtered through 0.22 µm nylon syringe filters (VWR, Germany).

For ectoine extraction, a 2.0 mL culture sample was centrifuged (Z 383 K, Hermle Labortechnik GmbH, Germany) at 5,000 rpm and 4 °C for 5 minutes. The supernatant (1.8 mL) was discarded, and the remaining pellet was extracted with 1 mL methanol by resuspending the pellet. Following a second centrifugation step under the same conditions, 0.4 mL of the methanolic extract was transferred into a new tube and evaporated overnight at 55 °C in a drying cabinet. The dried residue was then resuspended in 1 mL deionized water and incubated for 30 minutes in a thermomixer (Thermomixer Comfort, Eppendorf SE, Germany) at 1,000 rpm and room temperature. After a final centrifugation at 10,000 rpm and 4 °C for 20 minutes, 0.1 mL of the supernatant was transferred to a vial and diluted 1:10 with acetonitrile for analysis.

Ectoine samples were analyzed on a Vanquish UHPLC coupled to an Exploris 240 mass spectrometer with an OptaMax NG ion source (Thermo Fisher Scientific, Germany). Solvents and modifiers used were of Honeywell LC-MS grade. An **Agilent Poroshell 120 HILIC-Z 2.1x150x2.7µ** column with guard was used for separation in a 20 min run at 0.4 ml/min, using the following binary linear gradient conditions: 0 min 100% B, 9 min 82% B, 10 min 45% B, 11 min 45% B, 12 min 100% (A - 10 mM ammonium formate pH 3.0 in 2% aq. acetonitrile, B - 10 mM ammonium formate pH 3.0 in 92% aq. acetonitrile). Injection volume was 1 µl and column temperature was 25°C. MS parameters were as follows: heat-supported electrospray ionization, positive mode at 3.5 kV and 320°C; gases (sheath, aux and sweep) at 50, 10 and 1 arb. units; transfer tube temperature 300 °C; MS^1^ full scan detection 55 – 825 m/z, 60k orbitrap resolution, 3 microscans averaging, AGC target ‘Standard’, max. injection time 100 ms. Ectoine was quantified in extracted ion chromatograms of 143.08150 m/z (5 ppm tolerance, [M+H]^+^) upon a calibration series of ectoine in acetonitrile, ranging from 0.5 µM to 30 µM.

Ectoine eluted at 9.0 min as a well-defined peak; however, nearby eluting peaks of residual or background salt ion clusters were also detected. We therefore assessed the approximate ion suppressive effects using post-extraction spiking experiments. A correction factor of ~1.5 (averaged over 10 samples, standard deviation 0.18) was determined and applied to raw ectoine concentrations. Approximation of ion suppression effects was carried out by assessing signal increases after ectoine-spiking of volumes of pure acetonitrile (in triplicate) in comparison to 10 samples of time point 0 and 5, respectively. 198 µl of solvent or sample were spiked with either 2 µl of 300 µM ectoine standard (or 2 µl of acetonitrile for unspiked controls). By dividing the signal increase after spiking of pure solvent by the increases of the 10 spiked samples, and averaging those results, the ion suppression correction factor for the ectoine quantification method was estimated.

Gas composition was analyzed for the eCO_2_RR experiments using a gas chromatograph (INFICON AG, Switzerland) equipped with a thermal conductivity detector (TCD), using helium and argon as carrier gases. Gas samples were drawn through Tygon® tubing and the outlet was connected to a mass flow controller (EL-FLOW Prestige, Bronkhorst High-Tech B.V., Netherlands), operated via a Flow-Bus interface. Quantification was conducted via external calibration with certified gas standards for H_2_, O_2_, N_2_, CO_2_, and CO (R² = 0.99 for all analytes).

OD_600_ was measured using a UV-Vis spectrophotometer (Libra S11, Biochrom Ltd, UK) to monitor growth. At each sampling point during microbial growth and ectoine production experiments, pH was measured using a pH meter (LAQUAtwin, Horiba, Japan).

**1.5. Data Processing and Calculations**

Coulombic efficiency ($CE$) for formate, carbon monoxide (CO) via electrochemical CO_2_ reduction and hydrogen (H_2_) generation via hydrogen evolution reaction (HER) was determined (Eq. S1) by comparing the charge theoretically needed to produce the detected product amount ($Q_{product}$) with the total charge supplied over the course of the reaction ($Q_{total}$). Product quantification ($n_{product}$) was performed using HPLC for formate and gas chromatography for CO and H_2_. $CE$ calculation incorporated the measured product concentrations at each sampling point and the corresponding cumulative charge. A two-electron transfer ($z=2$) was assumed for formate (Eq. S2), CO (Eq. S3), and H_2_ (Eq. S4), and Faraday's constant ($F$ =96,485 C mol^−1^) was used.

$CE\left( \% \right)= \frac{Q_{product}}{Q_{total}} = \frac{n_{product} \times z \times F}{\int_{t=0}^{t} Idt} \times100$ Eq. (S1)

$$CO_{2} +H_{2}O+2e^{-}\to HCOO^{-}+OH^{-}$$

$E_{CO_{2} / HCOO^{-}}^{^{\circ}} = -0.64 V vs\mathrm{SHE}$ Eq. (S2)

$CO_{2} +2H^{+}+2e^{-}\to CO+H_{2}O$
$E_{CO_{2} / CO}^{^{\circ}} = -0.10 V vs \mathrm{SHE}$ Eq. (S3)

$$2H^{+}+2e^{-} \to H_{2}$$

$E_{H^{+}/H_{2}}^{^{\circ}} = 0 V vs \mathrm{SHE}$ Eq. (S4)

Ectoine yields ($\eta_{ectoine/formate}$,$\eta_{ectoine/methanol}$, mmol mol^−1^) were determined by relating the increase in ectoine content over a 5-day interval to the amount of substrate consumed during that period. Substrate consumption was corrected for evaporation and volatilization losses based on rates from abiotic controls as described in Section 2.5 of the main text. For formate, the amount of substrate consumed was calculated by correcting the measured change in formate content ($\Delta n_{formate}$, mol) with change derived from evaporation and volatilization effects ($\Delta n_{formate,volatilized}$, mol) (Eq. S5). The ectoine yield was then obtained as the ratio of the ectoine content increase to the corrected amount of consumed formate carbon (Eq. S6). Ectoine yields normalized to carbon input (mc-mol c-mol^−1^) was based on the amount of carbon per molecule in ectoine (6-C), formate (1-C), methanol (1-C), and for values reported in literature with glucose (6-C) and glutamate (5-C). Substrate-specific abiotic losses were determined from abiotic controls by linear regression of concentration over time. For formate, a daily loss rate of 0.0327 mM d^-1^ was obtained (R² = 0.97), corresponding to 1.18% per day. For methanol, the daily loss rate was 0.0779 mM d^-1^ (R² = 0.97), equivalent to 3.18% per day. Calculations for methanol followed the same formula structure, applying the respective carbon content and evaporation/volatilization correction.

$\Delta n_{formate, consumed}= \Delta n_{formate}- \Delta n_{formate, volatilized}$ Eq. (S5)

$\eta_{ectoine/formate} \boldsymbol{(}\mathrm{mmol} {mol}^{-1})=$ $\frac{\Delta n_{ectoine}}{\Delta n_{formate, consumed}}$ Eq. (S6)

**2. Results and Discussion**

**2.1 Preliminary Growth on Formate with *M. halotolerans***

Formate fed to *M. halotolerans* at the concentration at 15 mM was depleted under all tested conditions within 10 days, except for the initial seed culture derived directly from cryostock, prompting an increase to 20 mM in subsequent cultivations. At this concentration, complete substrate consumption was achieved (Figure S1). However, a short lag phase was observed in cultures supplemented with a defined vitamin solution, whereas those supplemented with yeast extract showed immediate formate uptake. In later experiments using ISR=0.16, both supplementation strategies supported stable substrate utilization, suggesting physiological adaptation to the elevated formate level. This transient lag-phase points to a potential threshold concentration at which formate may exert metabolic stress or require regulatory adjustment for efficient assimilation. Notably, ISR=0.08 cultures supplemented with vitamins showed a delayed uptake, with maximum consumption occurring between days 5–10. These values reflect condition-specific metabolic adaptation and potential bottlenecks in substrate assimilation pathways (Table S1).

To evaluate whether higher formate concentrations increase growth, cultures were fed with 100 mM formate. However, no growth was observed and only approximately 2.4% formate was consumed in inoculated samples compared to abiotic controls, confirming a strong inhibitory effect at this concentration. In prokaryotes, formate toxicity is often linked to the inhibition of terminal respiratory cytochromes, an effect that can be intensified by the passive influx of undissociated formic acid leading to cytoplasmic acidification and disruption of the proton motive force [57–58]. The concentration at which growth inhibition occurs varies across species and is influenced in part by the activity of formate dehydrogenase. *Escherichia coli* LY01 exhibited complete growth inhibition at 17.5 mM formate (at pH 7.0) [59], while *Acidithiobacillus ferrooxidans* was unable to grow at 20 mM (at pH 1.8) [60] and *Thiobacillus sp.* A2 showed growth up to 25 mM but was inhibited at concentrations above 40 mM (at pH 7-8) [61].

To overcome formate toxicity at higher concentrations, a semi-continuous feeding strategy was implemented in which 20 mM formate was added every 10 days up to a cumulative input of approximately 100 mM (Figure S2). This approach enabled repeated substrate consumption and gradual biomass formation, indicating that *M. halotolerans* can tolerate high cumulative formate exposure, if maximum concentrations remain low.


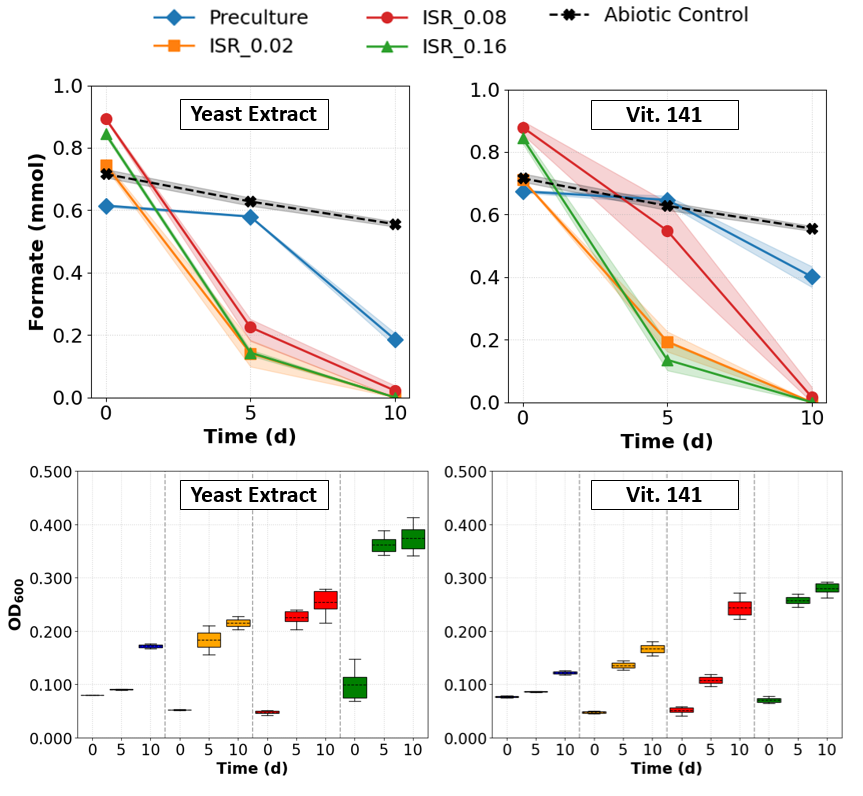


**Figure S1.** Formate consumption (top) and cell growth (OD_600_, bottom) of *M. halotolerans* at different inoculum-to-substrate ratios (ISR) in shake flask cultivations and abiotic control for formate at 30 °C. The left column shows experiments with yeast extract, the right column those with Vitamin 141 solution. Formate concentrations are shown as mean values with shaded areas (min. to max.). OD_600_ values are shown as boxplots (mean line, min. to max. as whiskers). (n = 2 for Seed Culture, ISR=0.02; n = 3 for ISR=0.08, ISR=0.16, Abiotic Control)


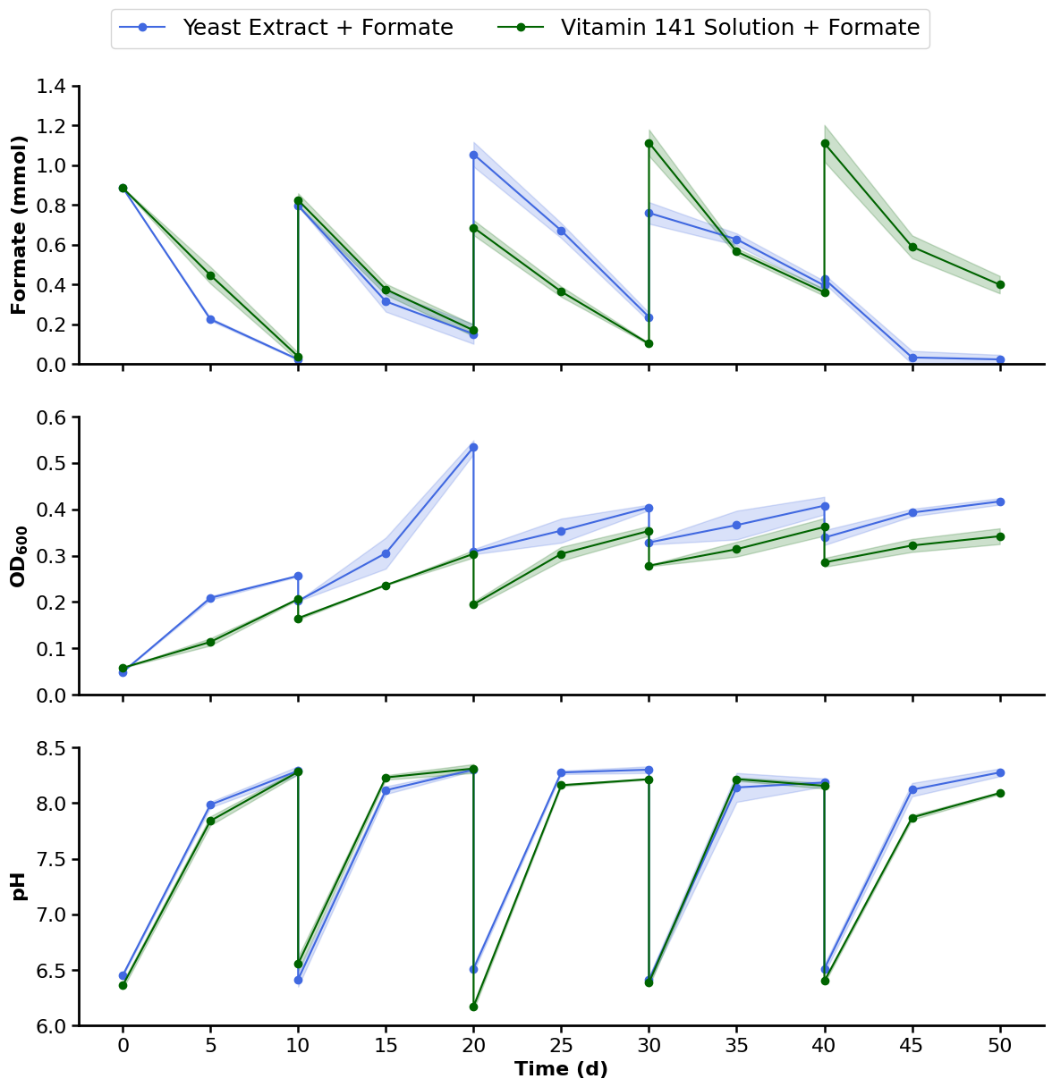


Figure S2. Semi-continuous formate feeding over a 50-day period for *M. halotolerans* cultures supplemented with either yeast extract (blue) or vitamin 141 solution (green). The top panel shows formate consumption, the middle panel displays cell growth (OD_600_), and the bottom panel depicts corresponding pH fluctuations. Formate was re-added every 10 days, with total culture volume adjusted to 50 mL and pH corrected using 0.2 N NaOH or 0.2 N HCl. The blue arrow marks the time point at which additional yeast extract, rather than formate, was added due to incomplete substrate consumption (n = 2).

**Table S1.** Formate utilization rates (mmol d^−^**^1^**) of *M. halotolerans* for yeast extract and Vitamin 141 supplemented cultures for preliminary growth studies.

| **Group** | **Maximum formate utilization rate (mmol d^−1^) with Yeast Extract** | **Maximum formate utilization rate (mmol d^−1^) with Vitamin 141** |
| --- | --- | --- |
| **Seed Culture** | 0.007 ± 0.001 | 0.005 ± 0.003 |
| **ISR**=**0.02** | 0.121 ± 0.007 | 0.103 ± 0.007 |
| **ISR**=**0.08** | 0.134 ± 0.006 | 0.066 ± 0.020 |
| **ISR**=**0.16** | 0.140 ± 0.002 | 0.142 ± 0.010 |

**2.2 Ectoine Production with C_1_-substrates**

Ectoine levels correlated with substrate consumption (Figure S3) being generally decreasing as formate or methanol was depleted. After 10 days, cultures were deliberately starved by 5 days withholding further substrate addition to verify full utilization. This strategy was required due to dilution steps needed to perform metabolite quantification under high salinity (9% NaCl). Additionally, after 5^th^ day of first feeding and during this starving period (between 10^th^ and 15^th^ days) consumption of ectoine content decreased for all groups similar to the inoculum control. This suggests that once the supplied substrate was decreased, ectoine was no longer synthesized but instead surplus was consumed, most likely as a maintenance resource under carbon limitation, consistent with the reported ability of halophiles to reutilize compatible solutes [62–63].

The biosynthetic conversion of C_1_-substrates to ectoine proceeds via the formation of oxaloacetate and acetyl-CoA, each requiring a distinct set of precursors and cofactors (Figure S5). Based on stoichiometric analysis, the production of one ectoine molecule from formate consumes a total of 17.5 formate equivalents: 7.5 formate are used for oxaloacetate synthesis releasing 3.5 CO_2_. 5.5 formate are used for acetyl-CoA synthesis releasing 3.5 CO_2_. Subsequently, 4.5 formate are fully oxidized to provide 3 NAD(P)H and 3 ATP for ectoine synthesis from oxaloacetate, acetyl-CoA and 2 NH_3_, reflecting the energetic cost of amino acid activation, reduction, and condensation reactions. In total, 11.5 CO_2_ equivalents are released per produced ectoine. In contrast, methanol serves as a more carbon-efficient substrate. Ectoine production from methanol requires only 7 molecules, with a single CO_2_ released. This includes 3 methanol for oxaloacetate synthesis (alongside assimilation of 1 CO_2_), 2.2 methanol for acetyl-CoA (with release of 0.2 CO_2_), and 1.8 methanol fully oxidized for ectoine synthesis from the precursors and NH_3_. The stoichiometric analysis presented here did not include a transhydrogenase reaction to convert NADH into NADPH. Such a reaction would be required to achieve full redox balance in the simplified model and in addition model did not account for additional energetic or metabolic costs such as transport processes, transhydrogenase activity, or cellular maintenance. Energetic cost for NH_4_^+^ uptake (e.g. via proton antiport) was also not considered, which would further increase the substrate demand.

Preculture grown on 100 mM methanol + 20 mM formate, used to inoculate the experimental sets, exhibited an ectoine content of 65.7 ± 0.2 µg ectoine/mg CDW at 10 days of cultivation. This value was lower than previously reported for *M. halotolerans* [16] which reached 180 µg ectoine/mg CDW when cultivated with 8.8% NaCl and 125 mM methanol. This was also below yields observed in other halophilic organisms or recombinant strains, such as *Halomonas elongata* BKAG25 with 206.4 µg ectoine/mg CDW [37], *Alkalibacillus haloalkaliphilus* with 170 4 µg ectoine/mg CDW [38], a recombinant *Escherichia coli* BL21 strain with 418 µg ectoine/mg CDW [39], and *Halomonas campaniensis XH26* strain with 351.13 µg ectoine/mg CDW [40]. The reduced accumulation may be attributed to the use of a minimally adapted culture, derived directly from a cryostock with only a single subculturing step, potentially limiting metabolic optimization for ectoine synthesis.

**Table S2.** Five-day interval changes (Δn_ectoine_, µmol) in *M. halotolerans* cultures grown under the indicated C_1_-substrates for net ectoine production (Δ>0, green shaded) or ectoine consumption (unshaded). Values represent mean ± SD based on three biological replicates (n = 3).

|  | **Δn_ectoine_ (µmol)** | | | | |
| --- | --- | --- | --- | --- | --- |
| **Group** | **t=0 / t=5** | **t=5 / t=10** | **t=10 / t=15** | **t=15 / t=20** | **t=20 / t=25** |
| **Inoculum control**  **(No substrate)** | − 2.490 ± 1.278 | − 1.367 ± 0.134 ^†^ | − 0.253 ± 0.188 | − 0.317 ± 0.044 | − 0.173 ± 0.026 |
| **20 mM formate** | 0.592 ± 0.121 | − 1.953 ± 0.291 | − 1.083 ± 0.255 | 0.019 ± 0.408***** | 0.077 ± 0.102***** |
| **20 mM e-formate** | −1.281 ± 0.116 | − 1.275 ± 0.248 | 0.645 ± 0.506 | − 0.275 + 0.118 | − 0.306 ± 0.104 |
| **20 mM methanol** | 5.910 ± 2.522 | − 2.394 ± 4.311 | − 4.158 ± 1.261 | 4.006 ± 0.500 | − 3.447 ± 0.633 |
| **10 mM methanol  + 10 mM formate** | 2.393 ± 0.855 | − 3.653 ± 1.572 | − 1.555 ± 0.835 | 3.409 ± 0.279 | − 2.878 ± 0.289 |
| † Only duplicate values were used at this sampling point, as one replicate in the inoculum control showed no detectable ectoine but ectoine was present at all later points.  * Within each condition, all replicates showed consistent directional change (either positive or negative), unless indicated by an asterisk (*), where individual replicate trends diverged. | | | | | |


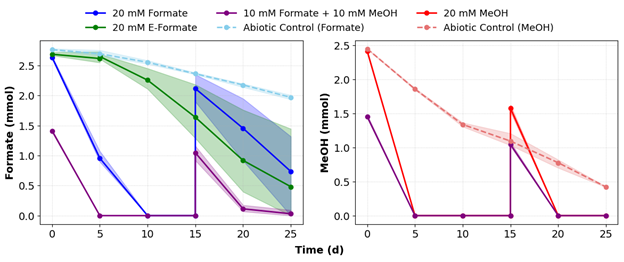


**Figure S3.** Time dependent concentrations of formate (left) and methanol (right) in cultures of *M. halotoleran*s grown in modified Choi medium with 9 % NaCl at 30 °C (n = 3). Substrate conditions include formate, e-formate, methanol, and co-substrate feeding of methanol and formate. Abiotic controls (no inoculum) and inoculum controls (no substrate) are shown for comparison (n=3).


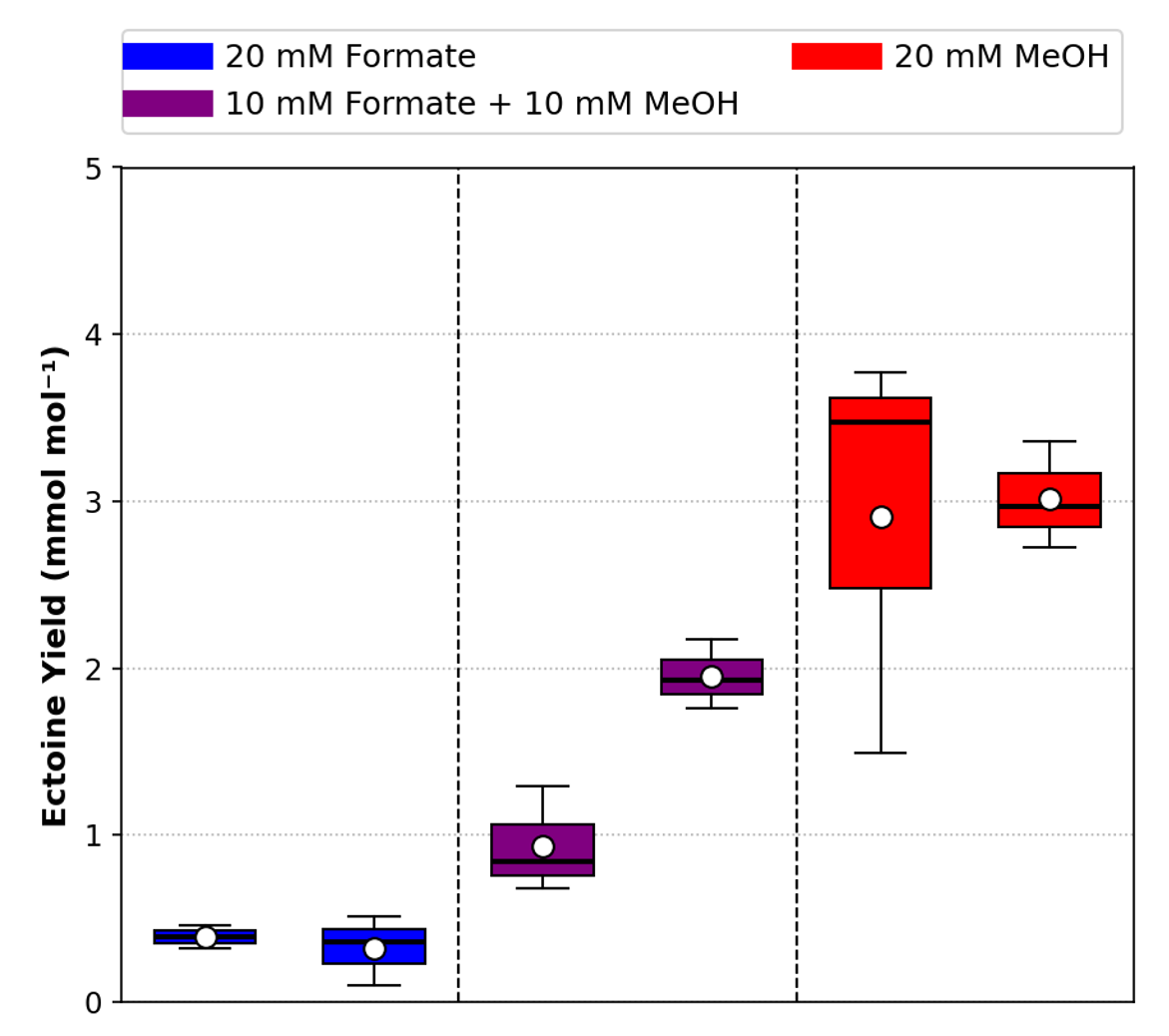


**Figure S4.** Substrate-specific ectoine yields ($\eta_{ectoine/substrate}$, mmol mol^−1^) achieved by *M. halotolerans* under different C_1_-substrate conditions. Comparison of yields from 20 mM formate, 10 mM formate + 10 mM methanol, and 20 mM methanol (n=3).


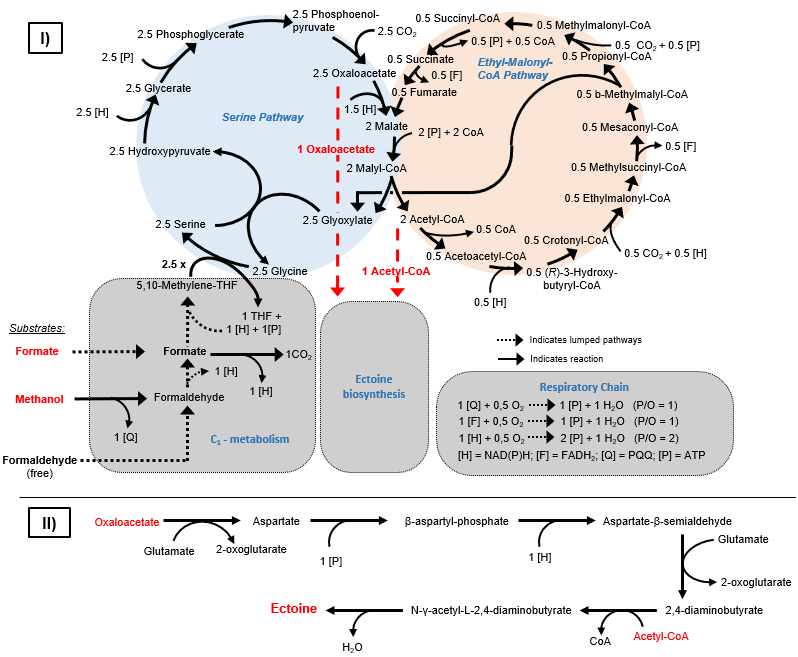


**Figure S5.** Stoichiometric and energetic requirements for ectoine biosynthesis from formate and methanol. (I) Substrate allocation toward oxaloacetate and acetyl-CoA formation from formate or methanol, including associated CO_2_ release. To take out 1 oxaloacetate and 1 acetyl-CoA (highlighted with red arrows), the serine cycle and the ethylmalonyl-CoA cycle have to run 2.5- and 0.5-times, respectively. (II) Reaction steps and cofactor demand for ectoine biosynthesis from oxaloacetate and acetyl-CoA using glutamate as N-donor. Assimilation of NH_3_ with 2-oxoglutarate to form glutamate is assumed to consume 1 NAD(P)H and 1 ATP (employing the glutamine synthetase/glutamate synthase system for NH_3_ assimilation).


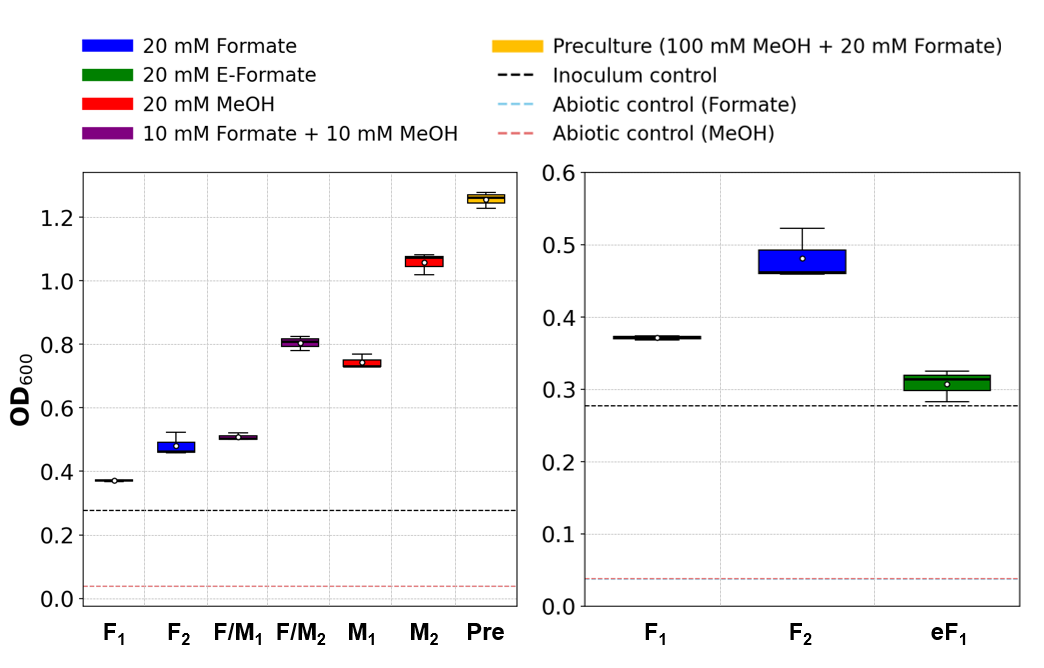


**Figure S6**. Final OD_600_ values of *M. halotolerans* cultures after growth in modified Choi medium with 9 % NaCl at 30 °C. C_1_-substrate conditions and preculture reference (left). Expanded view of formate and e-formate conditions (right). F, formate; F/M, formate + methanol; M, methanol; Pre, preculture and subscripts 1 and 2 indicate first and second feed, respectively. Abiotic and inoculum controls are included for comparison inoculum control (0.277 ± 0.006), the abiotic control (formate) (0.037 ± 0.001), and the abiotic control (methanol) (0.038 ± 0.001) (n = 3).

**2.3 Electrochemical Formate (e-formate) Production and Subsequent Microbial Utilization for Ectoine Synthesis**


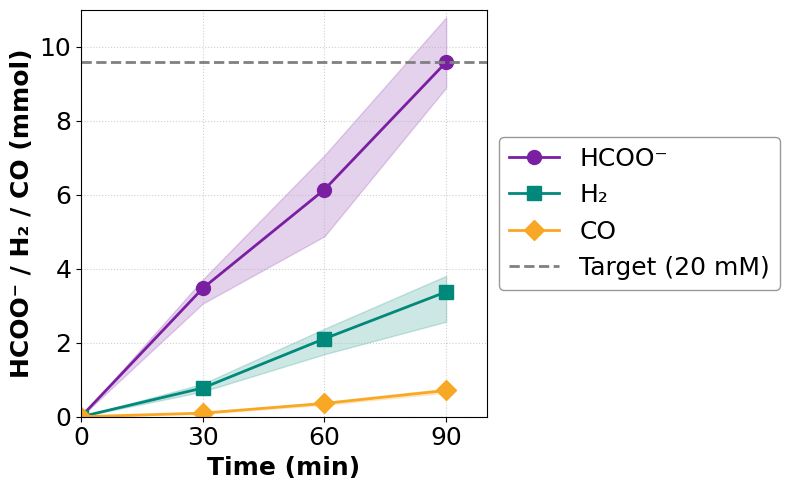


Figure S7. Time-dependent production of HCOO^-^, H_2_ and CO during eCO_2_RR at -50 mA cm^-2^ in modified Choi medium with 9 % NaCl supplemented with modified Vitamin 141 solution (n = 3).


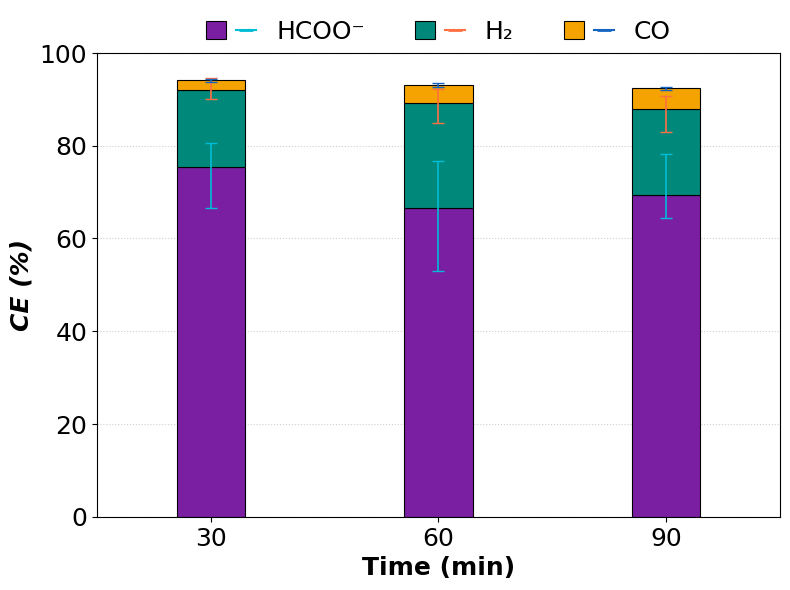


**Figure S8.**$CE$ for HCOO^−^ (purple), H_2_ (green), and CO (orange) for 30 min sampling intervals of eCO_2_RR at −50 mA cm^−2^ with modified Choi medium with 9 % NaCl supplemented with modified Vitamin 141 solution (n = 3).


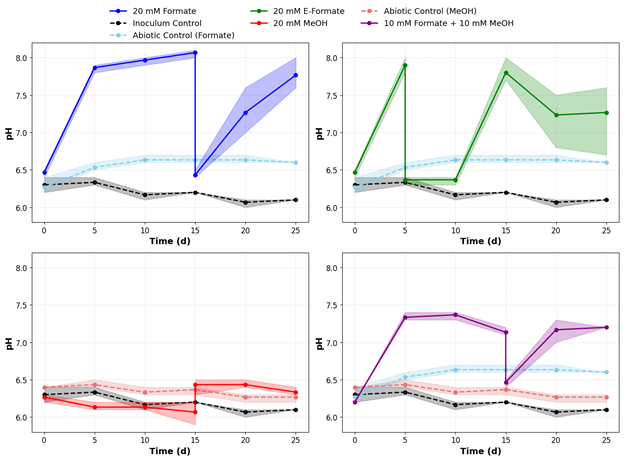


**Figure S9.** Time dependent pH values of *M. halotolerans* cultures grown in modified Choi medium with 9 % NaCl at 30 °C) under different C_1_-substrate conditions. Conditions include formate (top left), e-formate (top right), methanol (bottom left), and formate/methanol co-substrate (bottom right), with abiotic and inoculum controls included (n = 3).
